# An Integrated Approach for Assessing Aquatic Ecological Carrying Capacity: A Case Study of Wujin District in the Tai Lake Basin, China

**DOI:** 10.3390/ijerph8010264

**Published:** 2011-01-24

**Authors:** Chen Zeng, Yaolin Liu, Yanfang Liu, Jiameng Hu, Xiaogang Bai, Xiaoyu Yang

**Affiliations:** 1 Department of Land Resource Management, School of Resource and Environmental Science, Wuhan University, Luoyu Road 129, Wuhan, 430079, China; E-Mails: lunarzeng@gmail.com (C.Z.); yfliu610@163.com (Y.L.); hjm1210@163.com (J.H.); xiaogang880123@163.com (X.B.); ugbr4tnc2vh7@gmail.com (X.Y.); 2 Key Laboratory of Geographical Information System, Ministry of Education, Wuhan University, Luoyu Road 129, Wuhan, 430079, China

**Keywords:** aquatic ecological carrying capacity, indicator system, NDVI, GIS

## Abstract

Aquatic ecological carrying capacity is an effective method for analyzing sustainable development in regional water management. In this paper, an integrated approach is employed for assessing the aquatic ecological carrying capacity of Wujin District in the Tai Lake Basin, China. An indicator system is established considering social and economic development as well as ecological resilience perspectives. While calculating the ecological index, the normalized difference vegetation index (NDVI) is extracted from Moderate Resolution Imaging Spectroradiometer (MODIS) time-series images, followed by spatial and temporal analysis of vegetation cover. Finally, multi-index assessment of aquatic ecological carrying capacity is carried out for the period 2000 to 2008, including both static and dynamic variables. The results reveal that aquatic ecological carrying capacity presents a slight upward trend in the past decade and the intensity of human activities still exceeded the aquatic ecological carrying capacity in 2008. In terms of human activities, population has decreased, GDP has quadrupled, and fertilizer application and industrial wastewater discharge have declined greatly in the past decade. The indicators representing aquatic ecosystem conditions have the lowest scores, which are primarily attributed to the water eutrophication problem. Yet the terrestrial ecosystem is assessed to be in better condition since topographic backgrounds and landscape diversity are at higher levels. Based on the work carried out, it is suggested that pollutant emission be controlled to improve water quality and agricultural development around Ge Lake (the largest lake in Wujin District) be reduced.

## 1. Introduction

Carrying capacity has been applied to describe the importance of the limiting factors of environment on human material progress and to make the calculation and prediction of the upper limits of population or economic growth for a healthy eco-environment [1]. In the 1980s, widespread discussion about the drought problem initiated the employment of carrying capacity in resolving the constraint of water resources from which human society was suffering in China [2,3]. Simultaneously, rapid increases in nutrient input and environmental deterioration due to the intensified agricultural and industrial production around rivers and lakes have given rise to the applications of water environmental carrying capacity, which originated from the concept of environmental maximum load [4-6]. Recently, ecological or eco-environmental carrying capacity were highlighted as people became more aware of the need to detect and predict changes in ecosystem functioning in situations where human activities had profoundly affected ecosystems [7-9]. In order to integrate all the definitions of carrying capacity related to water, aquatic ecological carrying capacity was proposed in an attempt to assess the degree of pressure from human activities on ecological resilience.

Tai Lake region is one of the most densely populated and developed areas in China, situated in the lower reaches of the Yangzi River. During the past twenty years, rapid industrialization and urbanization, combined with a lack of effective management and technical measures, have caused many serious environmental problems such as water eutrophication, organic pollution and destruction of aquatic ecosystems [10,11]. Comprehensively, there are seven large- and medium-sized cities (namely, Shanghai, Suzhou, Wuxi, Jiaxing, Huzhou, Changzhou, and Kunshan) and 31 counties in the Tai Lake basin, in which Wujin District is located in the core of Changzhou city. The point pollution in this area is mainly attributed to dispersed rural industries such as township and village enterprises (TVEs), which were underpinned by the implementation gap in the toughest environmental laws [12,13]. The other prominent cause of water ecosystem degradation is non-point pollution triggered by intensive use of organic fertilizer (nitrogen, phosphorus) and other agricultural activities [14-16]. As a result, with the aim of evaluating the carrying capacity of the aquatic ecosystem in the context of diversified human activities and better protection of the aquatic environment, the National Environment Protection Agency in China initiated the “Water Special” project, in which aquatic ecological carrying capacity assessment was carried out in the Wujin District in the Tai Lake basin.

In the past, many methods were explored for assessing water resource, water environment or ecological carrying capacity, in which the multi-indicator assessment approach was the most widely used [17-19]. It followed a common procedure of indicator selection, weight determination, indicator (variables) estimation and integrated assessment. But the limitation arose when it was attempted to identify and estimate indicators reflecting ecological resilience in aquatic ecological carrying capacity. Previous research mainly focused on water consumption and water environment loading, and ecological indicators were often ignored in assessments [3,20]. For better reflection of nutrient cycles and energy flows in aquatic ecosystems, indicators representing both aquatic and terrestrial ecosystems should be incorporated [21,22]. With the accessibility of remotely sensed images and wide use of geographical information system, ecological indicators were capable of being quantified and the assessment of aquatic ecological carrying capacity could be more efficient and comprehensive [11,23,24].

This paper proposes an integrated approach to assess aquatic ecological carrying capacity of the Wujin District in the Tai Lake basin using MODIS (Moderate Resolution Imaging Spectroradiometer)- NDVI (Normalized Difference Vegetation Index) time-series images and GIS technology. Firstly, an operational indicator system was established in terms of human pressure and ecological resilience. Next, the integration of Delphi and the AHP (Analytic Hierarchy Process) was used to determine the weight of each indicator in different layers. Then based on empirical equations, indicators were calculated using statistical data, field data, land use maps and MODIS-NDVI time-series products. Finally, a comprehensive assessment of aquatic carrying capacity was performed based on defined criteria from 2000 to 2008, producing a final result for decision makers.

## 2. Materials and Methods

### 2.1. Study Area

**Figure 1:**
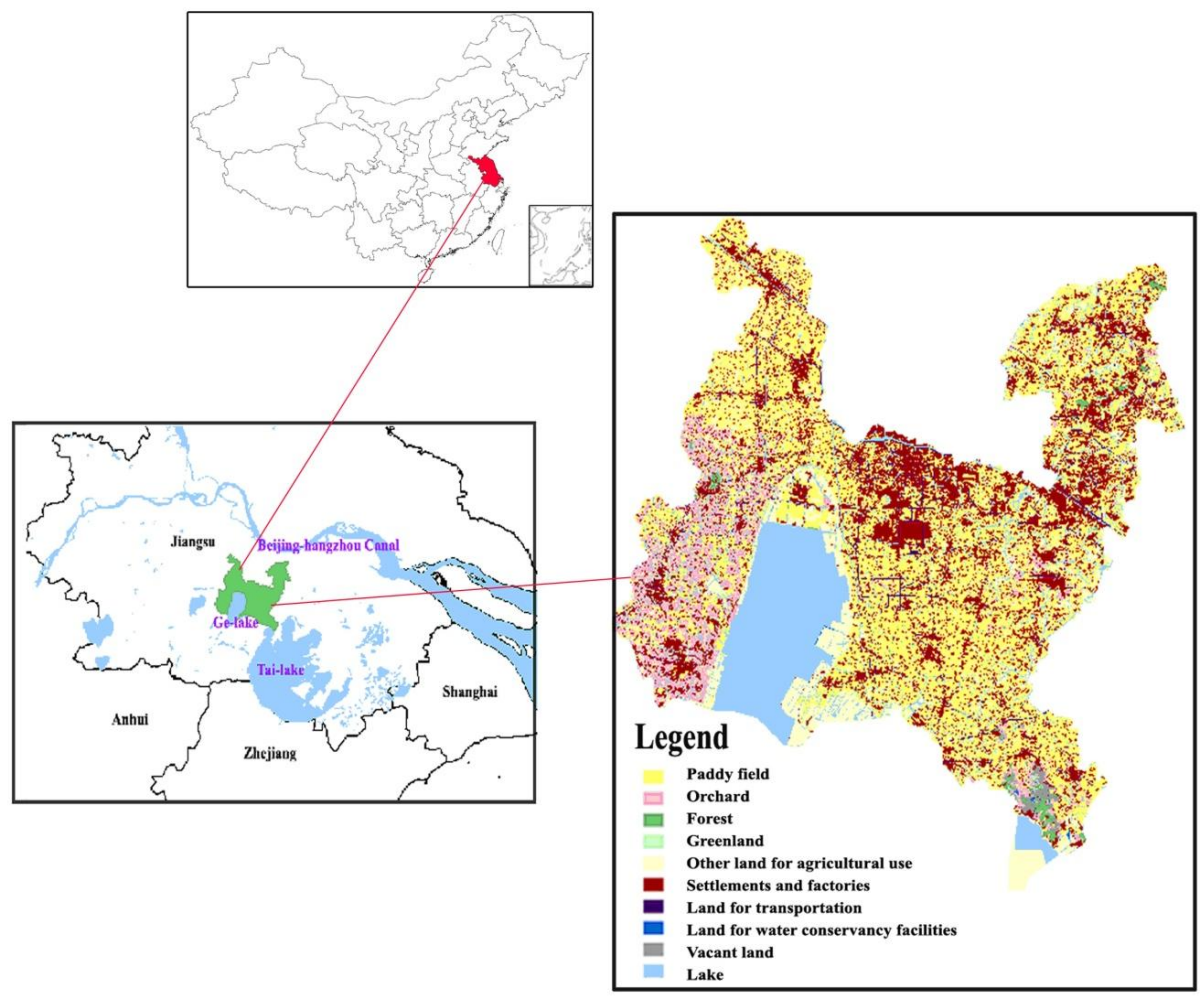
Location of the study area.

Wujin District is situated in the northwestern part of Tai Lake basin, in southeast China, with a geographical area of 1,246.6 km^2^. It links with Tai Lake, the third-largest freshwater lake in China and Beijing-Hangzhou canal, the oldest and longest canal in China (Figure 1). Rivers and lakes cover 23% of the total territory of Wujin District. Ge Lake is the largest one and is regarded as the western Tai Lake in the basin. The whole hydrological cycle of the area is controlled mainly by means of canalization (Su Nan canal from east to west, Xin Meng canal from north to south) and pumping stations. It is a typical agricultural district with more than 40% of the land area covered by paddy field and where the area of settlements and factories account for over 20% of the total area.

There are 14 villages and two economic and technological development zones (ETDZ) in Wujin District. As one of the most developed districts in the Yangzi River Delta, it has a population of around 0.98 million and its current gross domestic product (GDP) amounts to 85 billion yuan, 70% of which is from industrial output (according to the Statistical Yearbook of Wujin District in 2008). This rapid economic development has increased water demand greatly and water quality has deteriorated in recent years due to various point and non-point pollution sources [14,25].

### 2.2. Data Description

Based on the holistic understanding of aquatic ecological carrying capacity, data were collected through a series of approaches:

1. MODIS NDVI time-series products: the monthly composite MODIS 250 m normalized difference vegetation index (NDVI) dataset spanning five growing seasons (January 2003 to December 2007) were created for the research area.
2. Field monitoring data: field data were obtained from Changzhou Conservancy Bureau, listing parameters describing monthly water conditions such as total phosphorous (TP), total nitrogen (TN) and chemical oxygen demand (COD) concentrations (January, 2007 to August, 2009).
3. Land use map: land use data for 2005 were obtained from the land use survey database, at the scale of 1:10000.
4. Hydrological network map: the distribution map of primary and secondary river channels was obtained from the hydrological network map of the whole of Changzhou city.
5. DEM data: elevation data and slope data were provided by ISDSP (International Scientific Data Service Platform) with the spatial resolution of 30 m (2009).
6. Statistical data: Statistical Yearbooks of Wujin District from 2000 to 2008 were obtained in which population and economic data were included.
7. Auxiliary data: other data collected included the local Water Conservancy Construction Report in the National Eleventh Five-Year Plan (2005 to 2020), the local Social and Development Planning Report (2005–2020), agricultural and aquatic development planning reports and so on.

### 2.3. Selection of Indicators

Previous studies on the evaluation of regional carrying capacity in marine areas, arid inland river basins and urban areas revealed two primary obstacles for sustainable water usage: inadequate water supply and environmental pollution [26-29]. Moreover, it has been proved that the impact of any nation on environmental sources and sinks is deemed to be the product of its population, its level of affluence, and the damage done by the technologies that support that affluence [30]. Therefore indicators representing population density, economic growth, water resource consumption and water environmental loading were indispensible in the evaluation. Additionally, research on aquatic ecological carrying capacity was part of a trend to focus on methodologies for linking terrestrial and aquatic ecological ecosystems. A high probability of changes in the regional water quality of lakes as a consequence of location-specific forest-cover change was suggested [22,31]. Landscape patterns and their dynamics represented the physical framework of processes determining the ecosystem’s equilibrium [21]. The structure and function of land-cover determined the habitat environment; food webs and meta-populations associated with ecosystems were embodied at a meso-scale. As a result, in order to incorporate ecological resilience into the whole system, the structure and function of aquatic and terrestrial ecosystems should both be taken into consideration.

**Figure 2:**
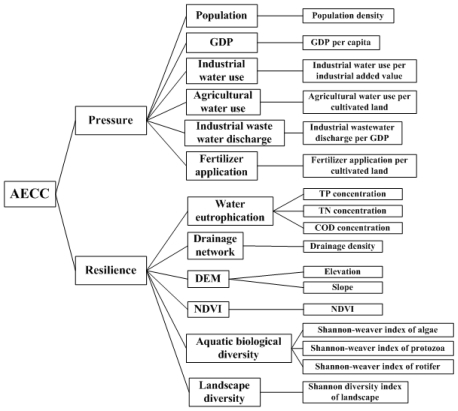
Indicator hierarchy for AECC assessment.

Taking all those factors into account, the established assessment model classified indicators into two categories: pressure and resilience (Figure 2). In the category of pressure, population, GDP, industrial water use, agricultural water use, and industrial wastewater discharge and fertilizer application were included. The specific indicators were calculated using data from the Statistical Yearbooks of Wujin District from 2000 to 2008. In the category of resilience, indicators of water eutrophication, drainage network, DEM, slope, NDVI, aquatic Shannon diversity and landscape Shannon diversity were included. The indicator of water eutrophication was characterized using parameters of TP, TN and COD concentrations. Field Samples indicating TP, TN and COD concentrations were used for Kriging interpolation in GIS [23]. Aquatic biological diversity was represented by the Shannon-Wiener index of plankton community, which reflects species richness, evenness of their distribution and their inter-correlations, and is considered the best measure of their joint influence [32]. Specifically, three groups consisting of algae (bacillariophyta, chlorophyta, cyanophyta, cryptophyta and others), protozoa (phytomastigina, zoomastigina, Amoebaproteus Pallas, ciliate) and rotifer (brachionus, keratella, asplanchna, polyarthra, filinia and others) were identified. The values of the Shannon-Weiner index in different groups were determined respectively by the average of the calculated values at different sample sites. Finally, the Shannon-Wiener index of plankton community was assessed by the sum of these three evenly weighted evaluated Shannon-Wiener indices. In accordance with the Land Use Classification System in China (2001 version), Shannon diversity of landscape was calculated using the area of 49 land use types extracted from the land use map of Wujin District.

### 2.4. Normalized Difference Vegetation Index Extraction

The nature of environmental problems required the use of tools able to capture changes in the structure and function of the ecosystems at large scales and a powerful description could be derived from the seasonal course of the NDVI [33-35]. It has been proved to correlate with green plant biomass and be sensitive to biophysical characteristics of vegetation such as canopy leaf area and net primary production [36,37]. Moreover, evaluating the potential of NDVI and NDVI-derived metrics for watershed monitoring and water quality studies was important in gaining an increased understanding of landscape-water quality relationships [38]. Consequently, MODIS NDVI time-series data were used in analyzing vegetation cover for aquatic ecological carrying capacity in the research area.

Preprocessing for MODIS was started with the emphasis on radiometric and geometric correction. Then NDVI time-series images were obtained through the operation of near infrared and red bands with regard to the following Equation (1). Next the MVC (maximum value composite) method was applied, in which the pixel observation with the highest NDVI value was selected to generate monthly NDVI products:

(1) $$NDVI=\frac{\rho_{NIR}-\rho_{red}}{\rho_{NIR}+\rho_{red}}$$

where *ρ_NIR_* referred to the pixel value on the infrared channel and ρ*_red_* referred to the pixel value on the red channel.

### 2.5. Integrated Assessment

A simple numerical scale related to the degree of aquatic ecological carrying capacity would seem a feasible way to convey findings in a comprehensive manner. Therefore, calculated indicators in the different categories mentioned above were transformed into a quantified grade scale between 0 and 1. In this judging set, the higher grade indicated a better condition in which a variable was capable of exerting beneficial effect on aquatic ecological carrying capacity. The final score of the aquatic ecological carrying capacity was calculated based on the quantified grades of indicators (variables) and their corresponding weights [Equations (2) to (4)]:

(2) $$V_{P}=\sum_{i=1}^{n}W_{pi}\times V_{pi}$$

(3) $$V_{R}=\sum_{j=1}^{m}W_{rj}\times V_{rj}$$

(4) $$AECC=W_{P}\times V_{P}+W_{R}\times V_{R}$$

where *V_P_* is the normalized value of pressure, *V_pi_* is the normalized value of indicators in the category of pressure, *W_pi_* is the respective weight of indicators in the category of pressure, *V_R_* is the normalized value of resilience, *V_ri_* is the normalized value of indicators in the category of resilience, *W_ri_* is the respective weight of indicators in the category of resilience and *W_P_*, *W_R_* are the weights of pressure and resilience, respectively.

The base criteria for aquatic ecological carrying capacity assessment was established according to national and local standards, books and literature, together with advice from experts on regional planning, water resource planning, land use planning, ecology, geography, *etc.* [9,39]. Meanwhile, the criteria were adapted in accordance with the quantitative analysis of sampled towns, counties, cities and provinces in the Middle and Southern parts of China. An integrated assessment criteria system was set up and the scores were classified into five levels, divided equally from 0 to 1 (Table 1). The weight of each index in different categories was obtained based on expert scoring and the AHP method.

## 3. Results and Discussions

### 3.1. NDVI Extraction and Analysis

**Figure 3:**
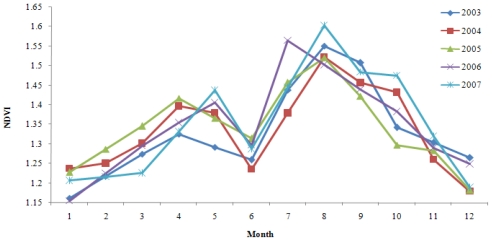
Temporal Variation in NDVI from 2003 to 2007.

**Figure 4:**
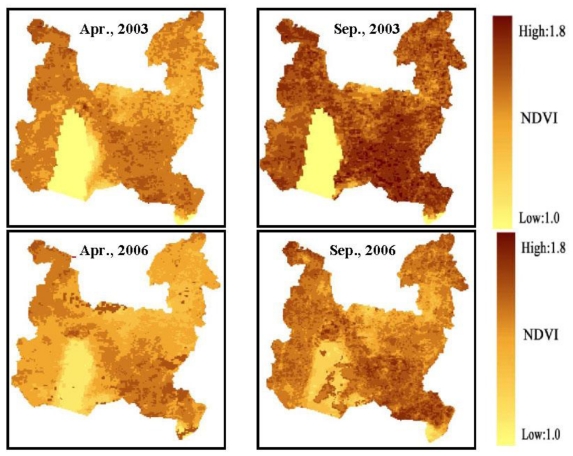
Maps of NDVI distribution. **(a)** April, 2003; **(b)** September, 2003; **(c)** April, 2006; **(d)** September, 2006.

According to the steps described above, the monthly NDVI distribution maps were generated and values were extracted for the period 2003 to 2007 in the Wujin District. It was shown that curves presented similar seasonal variation in different years from 2003 to 2007 (Figure 3). Two peaks in April or May and July or August could be observed in all curves, which demonstrated vegetation phenology. The value increased to 1.6 in autumn and decreased to 1.16 in early spring. It was estimated that the annual NDVI dataset were 1.328, 1.336, 1.343, 1.347 and 1.352, respectively, from 2003 to 2007, which meant the area of vegetation cover increased slightly. According to field investigation, vegetation in Wujin District consisted primarily of cropland, pasture, orchard and forest. In past years, industrial development had brought about the conversion of cropland to construction land; however, the farmland protection policy and the emphasis on forestry and the fruit industry guaranteed the area of orchard and forest in recent years, which could partly explain the slight upward trend of NDVI. In terms of spatial variation, the vegetation used to be more homogenous [Figure 4(a), (b)]. But now it covered a larger area in the southeast part of Wujin District, even with extension to Ge Lake, which demonstrated the dwindling of the lake area and the deterioration of water due to eutrophication. In the central and northeastern part of Wujin District, the value of NDVI decreased obviously, which could be attributed to the economic and technological development in the development zones. Green coverage in the northeast decreased and was substituted by villages according to the investigation [Figure 4(c), (d)].

**Figure 5:**
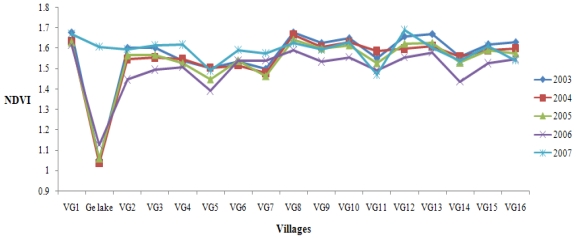
Spatial Variation in NDVI in August from 2003 to 2007.

For a better description of the spatial variation in NDVI and to discover where vegetation cover changed most from 2003 to 2007, the research area was divided into 16 VGs (14 villages and two economic zones) and one Ge Lake to make quantatitive analysis of NDVI in August (Figure 5). Significant changes in vegetation cover occurred in Ge Lake, especially in 2007. The value of NDVI rose to over 1.6 in 2007, which was attributed to the phenomena of cyanobacteria bloom and polder area construction. In recent years, deteriorating water eutrophication caused the rapid growth of algae, which had spectral features similar to terrestrial vegetation. Meanwhile, agricultural activities prevail in the polder area and vegetation cover has increased where cultivated land, pasture, and orchard exist. The vegetation cover was closely associated with water conservation, agricultural and ecological water use. Meanwhile, it was greatly affected by anthropogenic activities such as land use and, as a result, it was suggested that regulations be proposed on land use, in particular for the area around Ge Lake.

### 3.2. Integrated Assessment of Aquatic Ecological Carrying Capacity

In the past eight years, the population has decreased from 1,199,155 to 982,266 and the cultivated land declined by almost a half to 39,975 ha in 2008. However, GDP in 2008 was more than triple that in 2000, with more than 50% being contributed by industrial added value. Inevitably, the social and economic changes had a direct influence on agricultural and industrial water consumption, industrial wastewater discharge and fertilizer application. At the same time, due to the land use pattern change, irritation water use decreased from 632.16 to 280.42 million m^3^ and industrial water use increased from 127 to 150.52 million m^3^. Correspondingly, industrial wastewater discharge has doubled and fertilizer application presented a downtrend to 45.56 million kg so far (Table 2).

**Figure 6:**
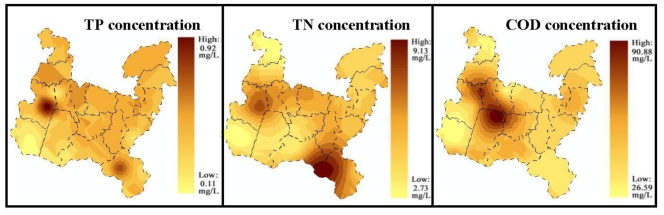
**(a)** Spatial distribution TP concentration (July in 2007); **(b)** Spatial distribution of TN concentration (July in 2008); and **(c)** Spatial distribution of COD concentration (July in 2007).

In terms of assessing ecological indicators, the Kriging interpolation approach was employed to interpolate COD, TP and TN concentrations in the whole research area to recognize the pollution source and to assess water eutrophication conditions. In the light of data availability and continuity, water samples indicating concentration of COD, TP and TN were selected and collected. After data input, processing and modeling in GIS, distribution maps of COD, TP and TN concentration were generated (Figure 6). Simultaneously, the maximum, minimum and mean values of COD, TP and TN concentration were recorded monthly (Table 3).

**Figure 7:**
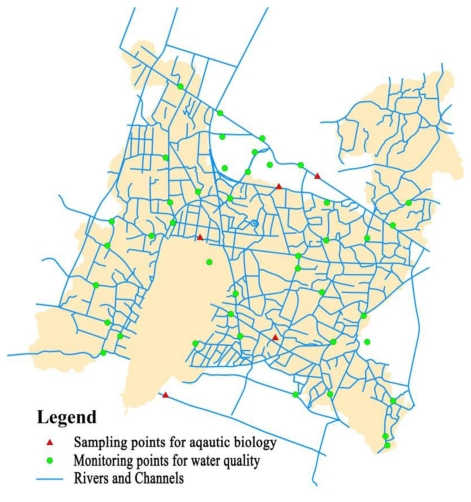
Hydrology network and sampling distribution.

As the map shows, the main pollution source was around the northwestern part of Ge Lake and the southeastern part of Wujin District (Figure 7). Through examination of the distribution map of sewage outlets, it could be determined that polluted areas were places where concentration of factories for chemical products, steel making, and electronic equipment existed. Furthermore, population aggregations around Ge Lake and in the southeastern part meant that domestic wastewater was an important component as well.

The calculated result of indicators of resilience is presented in Table 4. Resilience was defined in the literature as the capacity of a system to absorb disturbance and re-organize while undergoing change so as to still retain essentially the same function, structure, identity and feedbacks [40]. The concept of ecological /ecosystem resilience focused on persistence and robustness in the context of multiple equilibria and stability landscapes [41]. As a result, indicators of resilience were assumed to be stable over the past decade so as to make quantitative analysis of slow variables and fast variables for assessing their interactions in aquatic ecological carrying capacity.

The final result (Table 5) showed that the score of pressure increased from 0.35 to 0.57, which meant that human behaviors have been controlled, especially in agricultural activities. The amount of fertilizer application declined by more than half from 112.84 million kg to 45.56 million kg due to the loss of cultivated land and the improvements in fertilization technology. Meanwhile, the growth rate of industrial wastewater discharge decreased and the amount had been controlled to 88.5 million t in 2008. In terms of resilience, the concentration of TP, TN and COD have all exceeded the standard stipulated for healthy water and the Shannon diversity of plankton community in an aquatic ecosystem was extremely low, especially for the species of protozoa. In addition, topographic factors had important effects on hydrological and sedimentary processes and the result showed the low elevation and low topographic gradients of the area. It would decrease the risk of soil erosion and guarantee the stability of the integrated community within the aquatic and terrestrial ecosystems. The moderately diversified landscape and dense river network could be found from the resulting SHDI and drainage density. In the end, the aquatic ecological carrying capacity showed a slightly upward trend, increasing from 0.41 to 0.53. It was still at the third level, indicating that the intensity of human activities was exceeding the threshold of carrying capacity of aquatic and terrestrial ecosystems.

### 3.3. Discussion

Aquatic ecological carrying capacity assessment involved numerous factors including, but not limited to, population, resource availability, ecology, society, economics and government institutions [17]. The multi-index approach to the assessment was popular because it was capable of integrating relevant factors and variables as much as possible. Although a generic index system did not suffice for complete analysis of aquatic ecological carrying capacity, criteria were suggested to involve indicators from the perspectives of both human pressure and natural resilience. Based on the original definition of carrying capacity and the development of water resources, the water environment as well as the regional ecological carrying capacity, indicators selected herein included economic and social activities, ecological hydrology, structure and function of aquatic and terrestrial ecosystems, water resource utilization and water environmental loading.

Meanwhile, there was a tendency that problems in water resource management were reflected at a higher level such as the ecosystem level, and in a relationship with social and economic development. The evolution of water resources and water environmental carrying capacity to aquatic ecological carrying capacity was inevitable in sustainable development as it demonstrated how the natural ecosystem bears the burden of human development and makes a judgment on whether social and economic development should be constrained. As a result, it was recommended that aquatic, terrestrial ecosystem and human development be thoroughly embodied in aquatic ecological assessment in future work.

In addition, variation in the ecosystem itself was hard to measure and data availability made it even more difficult to handle in aquatic ecological carrying capacity assessment. However, with the increasing feasibility of using remotely sensed data, NDVI products with high temporal resolution could be helpful in assessing regional watershed conditions that affect water quality and stream condition [37]. Furthermore, spatial and temporal differences and their correlation could be observed through time-series NDVI maps, which would be beneficial for water management. In our research, NDVI was used as an important variable to indicate the structure and function of the ecosystem. It revealed that there was a slight upward trend annually in the past five years and similar seasonal fluctuation in each year. The distribution maps of NDVI indicated the vegetation extension around Ge Lake, which should be given close attention by the authorities. Furthermore, it was demonstrated that spatial analysis in ecological and resource management was capable of being incorporated at a higher level with the application of GIS technology [42]. In our research, Kriging interpolation was used to obtain the mean value of TP, TN and COD concentration and the areas of higher pollutant concentration were observed in the resulting maps. In the future, RS and GIS technologies are expected to be applied in aquatic ecological carrying capacity assessment in a wider and more profound way.

## 4. Conclusions

In our research, aquatic ecological carrying capacity assessment was implemented using RS and GIS in the Wujin District. The work concerned can be illustrated as:

1. Social, economic and ecological context was explicitly investigated in our case, formulating the background for the identification of indicators and the establishment of indicator hierarchy.
2. The spatial and temporal variation of NDVI was carried out and the extension of vegetation cover into Ge Lake was observed, which should be paid more attention by local authorities.
3. Ecological resilience assessment was made with the integrated consideration of aquatic and terrestrial ecosystems, in which RS and GIS technologies were of great assistance in obtaining the final result.

The eventual result of the integrated aquatic ecological carrying capacity assessment included all the indicators and their quantitative values. Also, maps were produced and spatial analyses were made accordingly. In the future, geo-spatial technologies should be incorporated in a more comprehensive, deep and quantitative manner into AECC. Generally, based on the work we have carried out, several suggestions could be made for decision makers.

1. Vegetation cover has increased in central and eastern parts of Wujin District in the past few years, which could be observed in NDVI products. However, the phenomena of cyanobacteria bloom and the decreasing of water area make it imperative to reduce agricultural activities around Ge Lake, since it will impair the aquatic ecological balance and aggravate water eutrophication.
2. Concentrations of COD, TN and TP were considerably higher than the national standards for pollutant emission; thus water eutrophication was still diagnosed as the main problem in Wujin District. As a result, it is suggested that pollutant emission reduction should be strengthened, especially for industrial waste water discharge and agricultural activities.
3. In the long run, aquatic ecological carrying capacity has increased slightly, which reveals that government planning on land use, economic growth, population growth and technological levels was able to improve water management and could be reasonably conducted.

## Figures and Tables

**Table 1:** Criteria system for the assessment. V

Level		V	IV	III	II	I
Score		0–0.2	0.2–0.4	0.4–0.6	0.6–0.8	0.8–1
Indicator	Unit	Criteria				
Population density	/km^2^	>1000	500–1000	400–500	200–400	<200
GDP per capita	10^4^ yuan per capita	<2	2–2.5	2.5–3.75	3.75–5	>5
Agricultural water use per cultivated land	10^6^ m^3^/ha	<30 or >142	30–60 or 112–142	60–69 or 103–112	69–73 or 99–103	73–99
Industrial water use per industrial added value	m^3^/10^4^ yuan	>100	56–100	45–56	31–45	<31
Fertilizer application per cultivated land	kg/ha	>1200	600–1200	525–600	300–525	<300
Industrial waste water per GDP	m^3^/10^4^ yuan	>25	13–25	10–13	7–10	<7
Concentration of TP	mg/L	0.9–1.3	0.2–0.9	0.05–0.2	0.01–0.05	0.001–0.01
Concentration of TN	mg/L	9–16	2–9	0.5–2	0.1–0.5	0.02–0.1
Concentration of COD	mg/L	40–60	10–40	4–10	1–4	0.15–1
Drainage density	km/km^2^	<0.5	0.5–2	2–4	4–6	>6
NDVI	/	<0.5	0.5–1.5	1.5–3	3–4.5	>4.5
Elevation	meter	>1000	500–1000	50–500	5–50	<5
Slope	degree	>25	10–25	5–10	1–5	<1
Aquatic biological Shannon diversity	/	<1	1–1.8	1.8–2.5	2.5–3.5	>3.5
Shannon diversity of landscape	/	<0.5	0.5–1.25	1.25–1.75	1.75–2.25	>2.25
(AECC) Aquatic ecological carrying capacity		The threshold of aquatic ecological carrying capacity assessment was 0.6 and scores above it mean the ecosystem is capable of bearing the human activities.				

**Table 2:** Values of pressure factors in 2000, 2004 and 2008.

Pressure Factors	Units	2000	2004	2008
Population	/	1,199,155	960,804	982,266
GDP	10^4^ yuan	2,339,228	3,887,695	8,501,987
Industrial added value	10^4^ yuan	1,260,405	2,477,723	5,556,690
Cultivated land area	ha	78,457	48,629	39,975
Irrigation water use	10^6^ m^3^	632.16	359.52	280.42
Industrial water use	10^6^ m^3^	127	136.79	150.52
Fertilizer application	10^6^ kg	112.84	67.48	45.56
Industrial wastewater discharge	10^6^ t	39.75	87.87	88.5

**Table 3:** Concentration of COD, TN and TP.

	January 2007	April 2007	July 2007	January 2008	May 2008	July 2008	May 2009	August 2009
COD Concentration								

Max	77.46	89.39	90.88	66.13	45.7	57.94	120.49	46.52
Min	43.46	31.84	26.59	33.51	26.76	19.84	29.01	25.24
Mean	62.1	49.27	45.02	44.86	37.15	36.62	43.65	38.43

TN Concentration								

Max	11.25	9.23	8.02	10.72	14.4	9.12	6.38	2.54
Min	5.19	5.57	3.13	4.85	1.59	2.73	3.46	4.12
Mean	8.31	7.24	6.55	7.85	6.58	5.05	5.14	3.36

TP Concentration								

Max	0.61	0.98	0.92	1.21	0.498	0.028	0.489	0.181
Min	0.093	0.19	0.11	0.042	0.105	1.063	0.221	0.539
Mean	0.296	0.402	0.43	0.392	0.296	0.304	0.353	0.31

**Table 4:** Results of indicators of resilience.

Indicator		Value	Description
Water eutrophication (pollutants concentration)	TP	0.35 mg/L	Water eutrophication condition is assessed by the sum of the evenly weighted three pollutants evaluated values
	TN	6.26 mg/L	
	COD	44.64 mg/L	
Drainage density (DD)		2.37 km/km^2^	DD = Length/catchment area
NDVI		1.55	Mean value of the research area
Elevation		5.59 m	Mean value of the research area
Slope		1.102 degree	Mean value of the research area
Aquatic biological diversity (Shannon diversity index of plankton community)	Algae	2.93	Aquatic biological diversity is assessed by the sum of the three evenly weighted evaluated Shannon index of planktons
	Protozoa	1.24	
	Rotifer	1.79	
Shannon diversity of landscape (SHDI)		2.18	It is calculated according to Land Use Classification System (2001 version) in China

**Table 5:** Results of aquatic ecological carrying capacity assessment.

	2000	2004	2008
Pressure	0.35	0.41	0.57
Resilience	0.47	0.47	0.47
AECC	0.41	0.44	0.53

Level	III	III	III
